# Progress in achieving SDG targets for mortality reduction among mothers, newborns, and children in the WHO South-East Asia Region

**DOI:** 10.1016/j.lansea.2023.100307

**Published:** 2023-10-29

**Authors:** Neena Raina, Rajesh Khanna, Shuchita Gupta, Chandani Anoma Jayathilaka, Rajesh Mehta, Sabyasachi Behera

**Affiliations:** a WHO Regional Office for South-East Asia, Delhi, India; b Formerly with WHO Regional Office for South-East Asia, Delhi, India; c Monitoring, Evaluation, Research & Learning, Delhi, India

**Keywords:** Sustainable Development Goal, Regional flagship, Maternal mortality, Newborn mortality, child mortality, Trend, Priority actions, WHO South-East Asia Region

## Abstract

As we reach midway towards the 2030 Sustainable Developmental Goals (SDG), this paper reviews the progress made by the WHO South-East Asia Region (SEAR) and member countries towards achieving the SDG targets for maternal, newborn and child mortality under the regional flagship initiative. Indicators for mortality and service coverage were obtained for all countries and progress assessed in comparison to other regions and between countries. Equity analysis was conducted to focus on the impact on marginalized populations. The article also informs about the priority actions taken by the WHO SEAR office and countries in accelerating reductions in maternal, newborn and child mortality. Moving forward, the region and countries must strategize to sustain the gains made so far and also address challenges of inequities, sub-optimal quality of care, newer priorities like stillbirths, birth defects, early childhood development, and public health emergencies and adverse effects of climate change on human health.

**Methods**

We conducted a comprehensive data compilation from a variety of published sources to address the diverse requirements for capturing policy and programmatic actions at both regional and national levels, encompassing inputs, processes, outcomes, and impact. This article predominantly incorporates materials from the WHO Regional Office of South-East Asia, including annual reports of the Regional Director, reports and recommendations from technical advisory groups (TAGs), reports from pertinent regional consultation meetings, and internal unpublished records of regional gatherings. To augment our information sources, we conducted a literature search, encompassing related articles and gray literature, and performed a library search for reports accessible from publicly available sources. Our primary databases included Medline through PubMed (https://pubmed.ncbi.nlm.nih.gov/) and the World Health Organization (WHO) institutional repository for information sharing (IRIS) available online (https://apps.who.int/iris/). We used search terms such as “WHO SEARO,” “maternal mortality,” “neonatal mortality,” “under-5 mortality,” “maternal health,” “child health,” and “South-East Asia Region” for the period spanning from 2014 to October 2023. Relevant articles aligned with the objectives of this manuscript were retained and reviewed, and desired information extracted by the authors.

## Introduction

The United Nations (UN) Sustainable Development Goals (SDGs), highlight good health and well-being as one of the 17 goals to be achieved by 2030.^1^ Under SDG 3, combating maternal mortality, and neonatal and child mortality have been prioritized as Targets 3.1 and 3.2 respectively. The global SDG target 3.1 calls for reduction of maternal mortality ratio (MMR) to less than 70 per 100,000 live births by 2030. For countries, the supplementary target is that their national MMR should be less than 140 per 100,000 live births by 2030. Additionally, countries that had an MMR of less than 420 in 2010 should reduce national MMR by at least two-thirds from the 2010 baseline by 2030.^2^ The SDG target 3.2 for under-5 mortality rate (U5MR) is to reduce to 25 or less per 1000 live births and for neonatal mortality rate (NMR) to reduce to 12 or less per 1000 live births by 2030. The targets for U5MR and NMR are the same for global and country level. Every Newborn Action Plan has also defined global and country level target for Stillbirth Rate which is to reduce it to 12 or less per 1000 total births by 2030.^3^

The maternal, newborn, and child health targets within SDG 3 demand immediate focus due to the declining progress observed in recent years. From 2015 to 2020, the global MMR reduced by only four points, moving from 227 to 223 maternal deaths per 100,000 live births. This figure remains more than three times higher than the 2030 target of 70 maternal deaths per 100,000 live births Similar decline in progress of both U5MR and NMR was observed with their average annual rate of reduction being halved compared to 2000–2015 period.^4^

The World Health Organization (WHO) South-East Asia Region (SEAR), comprising of 11 countries, accounts for 26% of the world’s population, including 25% of total annual births, 25% of the under-5 population, 28% of women in the reproductive age group (15–49 years), and 28% of the adolescent population.^5^ While two countries (MAL, THA) in the Region are upper-middle income, rest of the nine countries are either lower-middle-income or low income countries.^6^ During the Millennium Development Goal (MDG) era (1990–2015), the WHO SEAR made progress in reducing maternal and child mortality. Seven countries achieved their targets of U5MR reduction by two-thirds from the 1990 levels in 2015.^7^ The Region as a whole, however, reached its U5MR target of 39 per 1000 live births in 2016, i.e., one year later.^8^ The region could not achieve the MMR target, though, out of only nine countries globally that achieved this target, three were from SEAR (BHU, MAL, TLS).^9^

As we reach mid-way of the timeline for achieving the SDGs, it becomes pertinent to review the progress made by the region and the member states towards achieving the SDG-3 targets in the last 10 years (2014–2023) and identify important learning for course correction. The year 2014 coincides with the time when regional flagships were defined including the flagship on reducing preventable maternal, newborn and child mortality. The purpose of this article is to summarize the current status and progress in major coverage and mortality indicators across SEAR towards the SDG 2030 targets related to maternal and child health in the context of regional flagship. It also informs about the challenges and priority actions taken by the countries and region to accelerate progress toward these targets, including specific regional initiatives that supported the countries towards achieving them and those aimed at addressing the direct and indirect effects of the COVID-19 pandemic.

## Progress toward maternal, newborn, and child survival (2015–2022)

### Trends in maternal, newborn, and child mortality indicators

Across all WHO regions, SEAR had the maximum reduction in MMR of 78% from 525 per 100,000 live births in 1990 to 117 per 100,000 live births in 2020 (Table 1). Significant reduction was also seen for the U5MR which declined by 76% during the same period. The reduction in NMR was slower compared to that in MMR and U5MR, with 67% reduction observed from 1990 to 2021, which was notably lower than that achieved by other regions. However comparison of mortality rate reduction during the last decade, that is from 2010 onwards, shows that SEAR has achieved maximum reduction in all the three mortality indicators compared to other regions (Table 1).

**Table 1:** Trends in maternal mortality ratio, under-5, and neonatal mortality rate by WHO regions.

WHO regions	Maternal mortality ratio per 100,000 live births					Under-5 mortality rate per 1000 live births					Neonatal mortality rate per 1000 live births				
	1990	2010	2020	% reduction 1990–2020	% reduction 2010–2020	1990	2010	2021	% reduction 1990–2021	% reduction 2010–2021	1990	2010	2021	% reduction 1990–2021	% reduction 2010–2021
Africa	965	647	531	45	18	176	100	72	59	28	45	32	27	41	17
Americas	102	60	68	33	14	43	18	13	70	28	18	9	7	59	20
Eastern Mediterranean	362	231	179	50	23	104	60	45	57	25	44	32	25	43	20
Europe	44	15	13	70	14	31	12	8	76	35	14	6	4	71	34
South-East Asia	525	197	117	78	41	119	52	29	76	45	53	29	17	67	40
Western Pacific	114	49	44	61	10	52	18	12	78	35	27	9	6	79	39
Global	385	254	223	42	12	93	51	38	59	26	37	22	18	52	22

In order to compare progress for maternal, newborn, and child mortality, the MMR values were taken from 1990 to 2020 from the United Nations Maternal Mortality Estimation Inter-agency Group (UN-MMEIG),^10^ while the NMR and U5MR estimates were taken from United Nations Inter-Agency Group for Child Mortality Estimation (UN-IGME) from 1990 to 2021.^11^ These estimates are used globally for comparison between regions, countries and over a period of time as they apply a common estimation method in the interest of comparability.

**Fig. 1:**
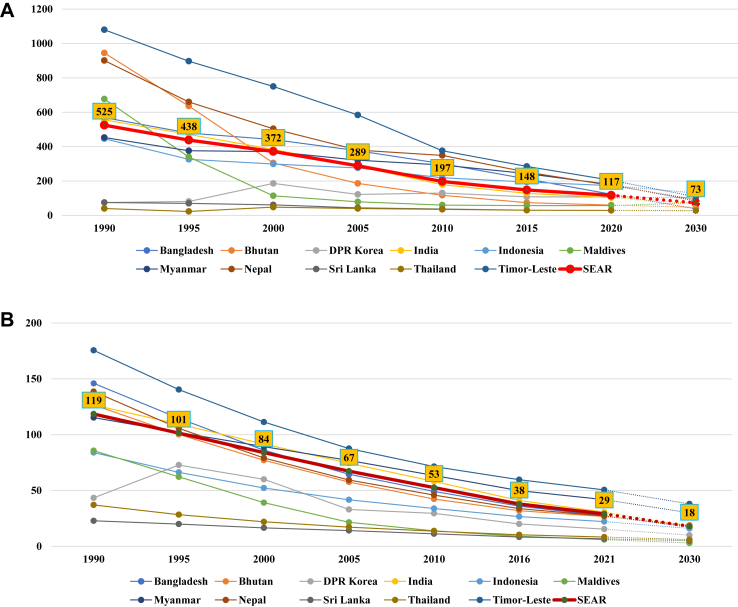

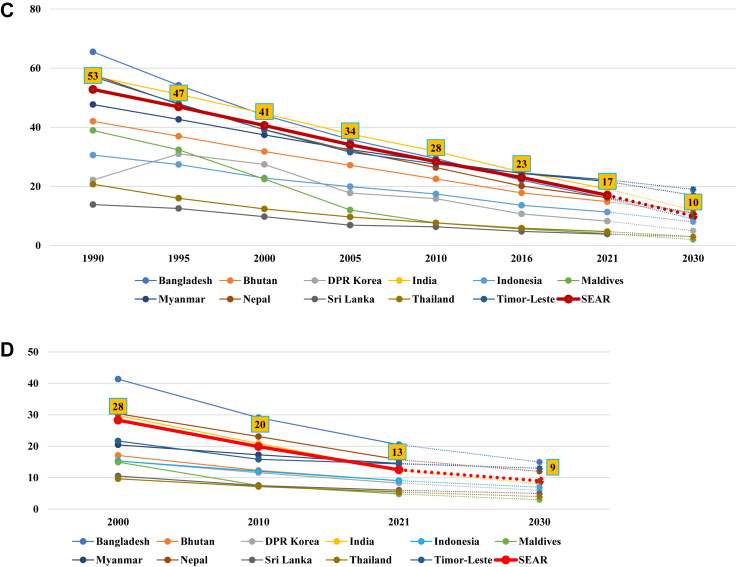
(**A**) Trends in maternal mortality ratio per 100,000 live births across different countries in WHO SEAR with projections to 2030^#^. ^#^Projections to 2030 calculated using annual reduction rate between 2016 and 2020 and assuming ARR is sustained till 2030; X-axis not drawn to scale. (**B**) Trends in under-5 mortality rate per 1000 live births across different countries in WHO SEAR with projections to 2030^#^. ^#^Projections to 2030 calculated using annual reduction rate between 2016 and 2021 and assuming ARR is sustained till 2030; X-axis not drawn to scale. (**C**) Trends in neonatal mortality rate per 1000 live births across different countries in WHO SEAR with projections to 2030^#^. ^#^Projections to 2030 calculated using annual reduction rate between 2016 and 2021 and assuming ARR is sustained till 2030; X-axis not drawn to scale. (**D**) Trends in stillbirth rate per 1000 live births reduction in WHO SEAR with projection to 2030^#^. ^#^Projections to 2030 calculated using annual reduction rate between 2010 and 2021 and assuming ARR is sustained till 2030; X-axis not drawn to scale.

The regional and country-specific trends for the mortality rates and stillbirth rate (MMR, U5MR, NMR, SBR) are shown in Fig. 1A–D. Countries within the SEAR show a variable picture in terms of mortality rates. As of 2020, seven countries have achieved MMR below 140 per 100,000 live births (BAN, BHU, DPRK, IND, MAL, SRL, THA), but none of the countries have achieved a two-third reduction from their 2010 MMR level. The country-specific projections show that four countries (BAN, Myanmar, NEP, TLS) are on track to achieve their national SDG target of two-third reduction in MMR from the 2010 baseline level (Fig. 1A). The scenario looks better for the under-5 mortality and neonatal mortality rates. As of 2021, five countries in the Region (DPRK, INO, MAL, SRL, THA) have achieved the SDG-3 targets for both the U5MR and NMR. For the U5MR and NMR, all countries are expected to achieve the SDG targets except Myanmar and Timor-Leste (Fig. 1B–C). The region as a whole will likely also achieve the SDG targets for U5MR and NMR, but narrowly miss the MMR target. As of 2021, six countries (BHU, DPRK, INO, MAL, SRL, THA) have achieved the 2030 stillbirth rate (SBR) target of 12 or less per 1000 total births. SEAR is on track to achieve the SDG target for SBR, though three countries (BAN, Myanmar, and TLS) will need to increase efforts to achieve it (Fig. 1D).

### Population-based coverage of essential maternal, newborn, and child health interventions

**Fig. 2:**
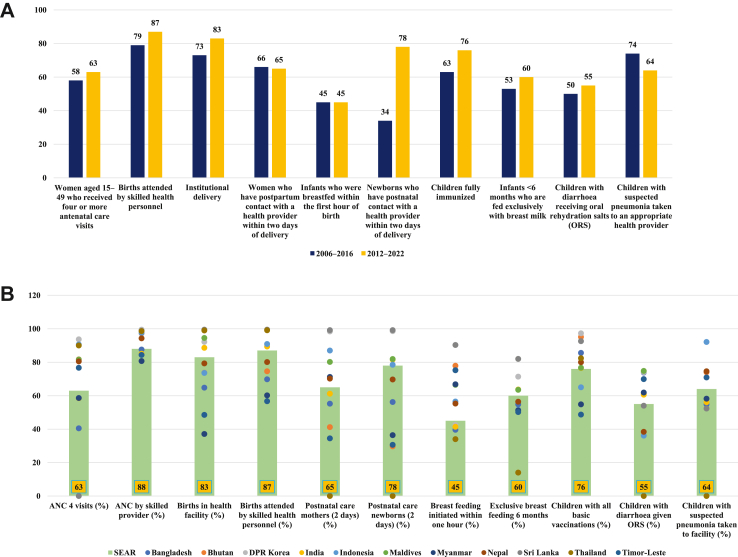
(**A**) Changes in regional coverage (%) of selected maternal, newborn, and child health interventions over time in WHO SEAR. (**B**) Country-level differences in the coverage (%) of essential key maternal, newborn, and child interventions in WHO SEAR.

The regional coverage of high-impact, evidence-based interventions across the Reproductive Maternal Newborn Child and Adolescent Health (RMNCAH) life-course was observed to have increased over time, but the increase has been uneven with large disparities within the countries (Fig. 2A). Almost two-thirds (63%) of women of reproductive age currently receive four or more antenatal visits, close to 90% of births are attended by skilled birth attendants, and the institutional delivery rate is high (83%). Nearly two-thirds of women (65%) have postnatal contact with a healthcare provider within two days after birth and less than half of the newborns were breastfed within the first hour of birth—both these indicators have shown no change over time. Newborns with postnatal contact with a healthcare provider within two days after birth has more than doubled from 34% to 78%. Less than two-thirds of infants less than 6 month of age are exclusively breastfed and only 76% of children fully immunized. The coverage of oral rehydration salts for children with diarrhea is 55% and close to 64% of children with suspected pneumonia are taken to an appropriate health provider (Table 2).

**Table 2:** Country-wise coverage of selected maternal, newborn, and child interventions over time.

Name of the countries	Year	Proportion of women aged 15–49 who received four or more antenatal care visits	Proportion of births attended by skilled health personnel	Institutional delivery	Proportion of women who have postpartum contact with a health provider within two days of delivery	Proportion of infants who were breastfed within the first hour of birth	Proportion of newborns who have postnatal contact with a health provider within two days of delivery	Percentage of children fully immunized	Percentage of infants <6 months who are fed exclusively with breast milk	Percentage of children with diarrhoea receiving oral rehydration salts	Proportion of children with suspected pneumonia taken to an appropriate health provider
Bangladesh	2017–2018	47	53	49	52	69	52	78	65	83	40
	2022	41	70	65	55	40	56	86	55	74	55
Bhutan	2010	77	65	63	NA	59	NA	NA	49	74	74
	2012	82	75	74	41	77	30	95	53	61	74
DPR Korea	2009	94	100	95	87	18	NA	NA	89	74	80
	2017	94	100	92	98	43	99	97	71	74	(∗)
India	2015–2016	51	81	79	65	42	27	62	55	51	78
	2019–2021	59	89	89	61	41	82	77	64	61	56
Indonesia	2012	88	83	63	80	49	48	40	42	39	75
	2017	91	91	74	87	57	79	65	52	36	92
Maldives	2009	85	95	95	67	64	NA	93	48	57	NA
	2016–2017	82	100	95	80	67	82	77	64	75	NA
Myanmar	2009–2010	NA	71	36	NA	76	NA	97	24	61	69
	2015–2016	59	60	37	71	67	36	55	51	62	58
Nepal	2014	69	58	57	57	55	57	78	66	37	87
	2022	81	80	79	70	55	70	80	56	38	75
Sri Lanka	2006–2007	93	99	98	91	80	91	97	76	51	58
	2016	NA	100	100	99	90	99	93	82	54	52
Thailand	2015–2016	91	99	99	NA	40	NA	72	23	73	80
	2019	90	99	99	NA	34	NA	82	14	NA	NA
Timor-Leste	2009–2010	55	30	22	25	82	NA	53	52	71	71
	2016	77	57	49	35	75	31	49	50	70	71
SEAR	2005–2016	58	79	73	66	46	34	62	53	50	74
	2012–2022	63	87	83	65	45	78	76	60	55	64

(∗) Means Figures that are based on fewer than 25 unweighted cases.

Mortality reduction is a function of scale and quality of implementation of evidence-based interventions at the population level. Country-level data of coverage indicators (Table 2) was obtained from the latest country-level Demographic Health Survey/Multiple Indicator Cluster Survey (DHS/MICS) reports available from each Member State (Supplementary Table S1).^12^, ^13^, ^14^, ^15^ The regional average was calculated from the population-weighted national coverages of the eleven member states. For interventions where standard global or regional or country-level data or estimates were not available, data were extracted from regional-level publications or databases, with due reference to source.

Significant variations were observed on comparing coverage data between the member countries (Fig. 2B). While eight of eleven countries (BHU, DPRK, INO, MAL, NEP, SRL, THA, and TLS) have achieved >75% coverage for ANC four visits and skilled birth attendance, only six countries (DPRK, IND, MAL, NEP, SRL, THA) have achieved >75% coverage for institutional deliveries. For the newborn and child indicators, eight countries (BAN, BHU, DPRK, IND, MAL, NEP, SRL, THA) have achieved >75% coverage for proportion of children fully immunized, five countries have >65% coverage of breastfeeding in the first hour of birth and six countries have PNC contact within first two days after birth >65%. For the older children, seven countries have >60% coverage of ORS for diarrhea and four countries have >70% for children with pneumonia taken to an appropriate health worker.

### Equity analysis

Equity in health care means “leaving no one behind” by ensuring that there should be no avoidable or remediable health-related differences among populations or groups defined socially, economically, demographically or geographically.^16^ Our analysis show that at the regional level, differentials in rates of institutional deliveries and skilled birth attendance (SBA) were most conspicuous when compared for maternal education and wealth quintiles but did not vary as widely when compared for rural and urban place of residence (Table 3). For early initiation of breastfeeding and for proportion of children with diarrhea receiving oral rehydration salts (ORS), the coverages were almost universally low, irrespective of the status of maternal education, wealth quintile or place of residence.

**Table 3:** Performance of South-East Asia Region on key RMNCAH indicators across equity profiles.

	BAN	BHU	DPRK	IND	INO	MAL	MYA	NEP	SRL	THA	TLS	SEAR
**Institutional delivery (%)**												
Maternal education												
No education (Pre-primary or none)	44.7	51.5	92.1	74.8	30.3	(95.5)	12.8	59.6	97.6	98.1	25.9	65.0
Secondary education (Secondary complete or higher)	80.6	93.1	92.6	96.9	87.9	92.4	82.6	93.1	98.9	99.8	91.2	93.9
Wealth quintile												
Poorest	42.4	33.2	82.7	76.2	45.4	96.8	16.8	65.8	99.5	97.5	16.7	68.0
Richest	87.4	95.8	97.6	97.4	93.9	88.8	82.5	97.6	98.8	99.2	87.2	95.7
Geography												
Urban	76.3	89.8	95.4	93.8	88.1	92.1	70.1	80.9	99.0	98.8	84.0	90.9
Rural	60.5	52.2	87.3	86.7	60.0	95.8	27.6	76.5	99.6	99.1	34.2	79.4
**Proportion of births attended by skilled health personnel**												
Maternal education												
No education (Pre-primary or none)	48.2	53.6	99.4	78.4	46.4	(100.0)	28.0	60.9	100.0	99.4	32.8	70.6
Secondary education (Secondary complete or higher)	85.8	93.6	100.0	95.8	98.0	100.0	94.8	94.3	99.8	99.8	94.6	95.4
Wealth quintile												
Poorest	47.0	34.3	98.9	79.3	74.5	100.0	36.3	67.0	99.2	97.9	26.2	75.3
Richest	90.5	95.1	100.0	96.8	98.9	99.1	97.0	97.4	99.7	99.2	90.1	96.7
Geography												
Urban	82.2	89.5	100.0	94.0	95.8	99.2	87.8	81.4	99.6	99.1	86.4	93.1
Rural	65.2	54.3	98.7	87.8	86.2	99.7	52.3	77.6	99.5	99.1	44.8	85.0
**Proportion of infants who were breastfed within the first hour of birth**												
Maternal education												
No education (Pre-primary or none)	34.1	56.8	43.9	36.9	62.2	∗	66.3	60.8	∗	38.1	77.6	41.5
Secondary education (Secondary complete or higher)	39.0	65.0	36.5	41.5	55.6	66.0	64.8	45.8	99.6	31.1	68.0	44.3
Wealth quintile												
Poorest	39.0	55.2	35.8	39.3	58.1	69.6	65.7	61.7	99.2	35.9	73.3	43.5
Richest	41.7	62.6	48.0	43.0	56.8	54.7	70.4	41.0	99.5	31.0	77.0	45.9
Geography												
Urban	38.0	61.3	44.4	44.2	56.7	56.9	69.8	50.5	99.4	29.6	73.6	46.5
Rural	40.0	58.1	39.5	40.4	56.3	72.2	65.8	62.5	99.5	37.1	75.9	44.1
**Children with diarrhoea receiving oral rehydration salts (ORS) (%)**												
Maternal education												
No education (Pre-primary or none)	∗	72.3	73.4	59.7	(22.4)	NA	54.3	38.1	∗	NA	69.8	55.5
Secondary education (Secondary complete or higher)	76.1	76.1	(78.4)	60.3	31.9	NA	∗	43.1	(60.0)	NA	75.7	59.0
Wealth quintile												
Poorest	67.0	69.1	72.8	59.3	31.6	NA	62.5	43.5	52.1	NA	62.2	57.3
Richest	79.8	78.0	75.7	64.1	31.2	NA	(63.1)	(45.3)	(64.2)	NA	73.2	62.2
Geography												
Urban	82.8	78.8	77.2	62.5	36.7	NA	67.1	39.4	(47.3)	NA	69.1	61.7
Rural	70.8	71.4	70.7	60.1	35.6	NA	60.7	36.2	55.3	NA	70.4	58.6

Bangladesh, Maldives, Myanmar, Sri Lanka: An asterisk indicates that a figure is based on fewer than 25 unweighted cases and has been suppressed.

DPRK (Maternal education): Figure is for upper secondary as information for ‘no education’ is not available in the report.

DPRK, Indonesia, Maldives, Myanmar, Nepal, Sri Lanka: Figures in parentheses ( ) that are based on 25–49 unweighted cases.

BAN: Bangladesh; BHU: Bhutan; DPRK: Democratic People's Republic of Korea; IND: India; INO: Indonesia; MAL: Maldives; MYA: Myanmar; NEP: Nepal; SRL: Sri Lanka; THA: Thailand; TLS: Timor-Leste.

Equity analysis was conducted for four maternal and child health indicators by disaggregating coverage data at country level based on three key equity parameters (maternal education, wealth quintile, residential location) to understand the extent of disparities.

Higher maternal education (secondary level or higher) was associated with a higher institutional delivery rate in all countries except in MAL. It was also associated with higher proportion of women who had SBA and a higher proportion of children with diarrhea who were given ORS in all countries. Women from richer wealth quintile were more likely to have an institutional delivery (except in MAL), higher proportion of SBA (except in MAL and THA) and higher proportion of children with diarrhea who received ORS (except in INO). Wealth quintile did not show an association with proportion of infants who were breastfed within the first hour of birth in a few countries. The rate of institutional delivery was higher for women from urban backgrounds as compared to those in rural backgrounds except MAL, SRL and THA. Urban background was also associated with a higher proportion of SBA and a higher proportion of children with diarrhea who received ORS (except in SRL and TLS) (Table 3).

## Priority actions to accelerate progress toward maternal, newborn, and child survival

Over the past 10 years (2014–2023), the SEA Regional office along with the countries has taken a number of strategic actions to not only sustain the gains in reduction in maternal and child mortality achieved during the MDG era, but to also further accelerate progress towards the SDG-3 targets.

### Political commitment and partnerships

The regional flagship areas were defined in 2014 that included a specific flagship on ending preventable maternal, neonatal and child mortality. In support of this, a regional summit of the UN H6 agencies, i.e., WHO, UNICEF (Regional Office for South-Asia and East and Asia Pacific Regional Office), UNFPA, World Bank, UNAIDS, and UN Women was organized in 2015 resulting in a joint UN statement pledging to work with the national governments to help strengthen their leadership and capacity to undertake time-bound action to end preventable mortality in the Member States.^17^ In 2016, the Regional Committee adopted a resolution, SEA/RC69/R3, reiterating the region's commitment to accelerate efforts for more inclusive and dynamic actions on women's, children's and adolescents' health after the release of the Global Strategy for Women's, Children's and Adolescents' Health (2016–2030).^18^ Political commitment was further mobilized during the Regional Meeting of Parliamentarians (2018) when it issued a ‘Call to Action’ for member countries to implement necessary strategies to accelerate actions for improving health of women, children and adolescents.

### Technical leadership and strategic guidance

The SEA Regional Office undertook country level analysis of the situation of maternal, newborn and child health to identify priority countries for providing technical assistance. Regional strategic frameworks and guidance were developed along with evidence-based strategies and interventions to guide strengthening of the national maternal, newborn, and child health programmes.^19^^,^^20^ The guidance was accompanied by ongoing technical support through regional meetings, workshops and technical support visits to country offices. Multiple regional meetings of programme managers and partner organizations were organized on technical areas of Maternal Newborn Child Health (MNCH) to review progress, disseminate technical updates, experience-sharing across the countries and identify key actions to accelerate progress towards the SDG targets. These meetings were also used to develop country plans for implementation and scale-up, and country-level engagement for adaptation of guidance to their specific context at both the policy and programmatic levels. A recent RMNCAH policy survey (2018–2019) reflects that most member states in the Region have favorable policies for maternal, newborn and child health areas aligned with the global guidance (Supplementary Table S2).^21^

The Regional Office constituted a Technical Advisory Group (TAG) on maternal, newborn, and child health in 2015 to strengthen the regional mechanism to support implementation of high-impact approaches and monitoring progress, and additionally a technical subcommittee on Sexual and Reproductive Health (SRH) in 2019. The TAG guided policies and strategies to improve maternal, child, and adolescent health and met annually to review the progress made by the countries and provided recommendations on the way forward. Such technical committees have now been established in most countries of the region (Supplementary Table S3).

### Improving quality of care

Poor quality of care is responsible for causing higher number of maternal and newborn deaths compared to non-utilisation of care as a cause.^22^ Taking quality improvement as a key strategy, SEAR developed the regional framework for improving the quality of care in RMNCAH in 2015 as well as an integrated assessment tool for measurement.^23^^,^^24^ Subsequently, countries were supported in preparing national strategies and plans for improving the quality of care which has resulted in all countries having a functional national cell for quality of care with quality improvement alliance/partnership and national standards (Table 4).^25^ The Regional Office introduced the Point of Care Quality Improvement (POCQI) approach in 2016 in collaboration with UN partners and academic institutes, and further supported the establishment of learning networks and a regional community of practice for POCQI implementation. Healthcare teams at the national and subnational levels have been trained to build their knowledge and skills in quality improvement. Currently, POCQI model is widely used across countries in SEAR and beyond.^26^

**Table 4:** Status of uptake of quality improvement at the country level in WHO SEAR.^23^

	BAN	BHU	DPRK	IND	INO	MAL	MYA	NEP	SRL	THA	TLS
National cell for quality of care was established and functioning	Yes	Yes	Yes	Yes	Yes	Yes	Yes	Yes	Yes	Yes	Yes
National QI alliance/Partnership established	Yes	Yes	No info	Yes	Yes	No	No info	Yes	No	No info	No info
National standards for MNH aligned with Global standards	Yes	Yes	No info	Yes	Yes	Yes	Yes	Yes	No info	Yes	Yes
National QOC plan prepared and existing QI initiatives absorbed	Yes	No info	No info	Yes	Yes	Yes	No info	No info	Yes	No info	No info

BAN: Bangladesh; BHU: Bhutan; DPRK: Democratic People's Republic of Korea; IND: India; INO: Indonesia; MAL: Maldives; MYA: Myanmar; NEP: Nepal; SRL: Sri Lanka; THA: Thailand; TLS: Timor- Leste.

### Maternal death or maternal and perinatal death surveillance and response (MDSR and MPDSR)

MPDSR or any form of maternal and/or perinatal death review or audit aims to improve health services and pre-empt future deaths. The regional office supported the countries in introducing and scaling up MDSR followed by MPDSR, including the development and update of national MPDSR guidelines and training packages in several countries Six countries (BAN, BHU, INO, Myanmar, NEP, TLS) have established MPDSR within their health systems as part of national programme. Collaborations were initiated between SRL and BHU and SRL and NEP to scale up MPDSR across these countries. As of 2019, nine of eleven countries have a national policy/guideline requiring maternal death notification within 24 h, all eleven countries have a national policy or guideline requiring a review of all maternal deaths, and ten of eleven countries have a national and all countries have a sub-national panel to review maternal deaths.^21^ In 2020, SEARO developed the first global virtual MDSR capacity-building training programme in partnership with Momentum (a USAID supported initiative) and later expanded as MPDSR which has been used to build health provider capacity in ten countries.^27^ Implementation of MPDSR has been identified as a regional key performance indicator for the Region.

### Monitoring and evaluation

The regional office is regularly monitoring the mortality and coverage data at the country and region level, and has developed regional/country fact sheets to help countries identify weak areas and strengthen national actions accordingly. A monitoring framework for RMNCAH programmes in the countries and the region was developed to harmonize essential and priority indicators in RMNCAH related metadata, frequency of measurement, disaggregation, and potential data sources to guide countries in strengthening data collection and monitoring mechanisms.^28^

### COVID-19 emergency preparedness and response

During the COVID-19 pandemic, the Regional Office assisted the member countries in continuing RMNCAH services through technical and operational guidance for modification of service delivery design and platforms, capacity development through virtual media, and research on the impact of COVID-19 on pregnant women and children. Under the global ‘Investment Project’, disruption of services was monitored using 34 routinely collected indicators through the Health Management Information system (HMIS) system in three countries—Bangladesh, Nepal and Timor Leste. There was a nearly two-fold drop (19.6%) in the proportion of births at health facilities in the year 2020–2021 as compared to the year 2019–2020 in Bangladesh. Similarly, there was a ten-fold drop in facility births in Timor Leste during the same time period. While there was a reduction in the percentage of newborns breastfed within 1 h of birth in 2020–2021 in Nepal (−6.3% to −11.7%), an increase from the baseline level of 2019–2020 was seen in Bangladesh. Number of newborns who received care within 24 h of births increased in Bangladesh (−20.0% to −0.1%) and Timor Leste (76.9% to −191.6%) and reduced in Nepal (−0.4% to −1.2%) for the period between 2019–2020 and 2020–2021 (Supplementary Table S4). In summary, though progress was noted with regard to utilization of several services as compared to 2020, health services especially maternal health services have not reached the pre-pandemic level (2019) yet.

## Newer Regional Programmatic Initiatives

### Prioritizing birth defects

Birth defects are estimated to cause 4.5% of newborn deaths and 3.2% of deaths of children between 1 month and 5 years of age globally.^29^ According to the WHO Global Health Observatory, contribution of birth defects as a cause of under-5 deaths in SEAR has increased from 6.2% in 2010 to 9.2% in 2019.^30^ The Region has supported the countries in establishing hospital-based surveillance mechanisms to measure and track the burden of birth defects, and in integrating it with the national routine health information systems. All countries of the SEAR have introduced rubella vaccination in the national immunization programme, with an average of 83% coverage. Maldives and Sri Lanka have eliminated rubella, and Bangladesh, Bhutan, Nepal, and Timor-Leste have controlled the occurrence of congenital rubella syndrome. Similarly, folic acid, which reduces neural tube defects is essentially given to all pregnant women. All countries are implementing folic acid supplementation, and several have fortified foods such as wheat flour with folic acid, vitamin B-12, and iron to prevent neural tube defects and anemia, though universal fortification is yet to become a reality. Use of antenatal ultrasound scans to detect physical anomaly in the fetus along with newborn screening for visible birth defects have been introduced, but the coverage needs to be expanded significantly and genetic and metabolic screening need to be introduced. Basic services for the care and management of children born with birth defects are available in most countries but advanced treatments, rehabilitation, and support for affected babies and families is limited.

### Improving family planning and safe abortion services

Contraceptive use is estimated to have averted 199,383 (80% CI: 146,136–360,747) maternal deaths in 2018 in the region, more than three-quarters (77.5%) of all expected deaths in the absence of any contraceptive use. An additional 16,854 maternal deaths (29% of the current level) could be averted by satisfying the current unmet need for contraception.^31^ In SEAR burden of unintended pregnancy is 58 per 1000 women of reproductive age (WRA), abortion rate is 46/1000 WRA and 74% of unintended pregnancies end in abortion. Additionally, unsafe abortions are responsible for 8% of all maternal deaths.^32^ Although the coverage and penetration of family planning and safe abortion services has improved in the region, challenges exist with regard to changing fertility desires, access to safe services, integrity of abortion supply and referral chains, data-driven accountability and program action, and stakeholder engagement.^33^, ^34^, ^35^, ^36^ The Region is actively addressing these issues through policy dialogue, supporting countries to adopt the latest guidelines, standards and recommendations, capacity-building activities like the training of government focal points and professional bodies in drafting policy briefs, conducting stakeholder mapping and data analysis, and supporting supply of commodities like injectable contraceptives in selected countries.

## Discussion

Since 2014, under the regional flagship initiative on accelerating reduction in maternal, newborn and child mortality, the WHO South-East Asia Region has given due emphasis to implementation of national plans with particular focus on evidence-based actions for high returns across the MNCH continuum. However, challenges remain due to variable progress within countries in terms of coverage of key RMNCAH interventions particularly socio-economic disparities, quality of health services, and availability of data for improved health outcomes. Another challenge is the emerging causes of mortality and morbidity (stillbirths, birth defects, disabilities, climate change, etc) especially among countries which have already achieved the SDG targets. This requires the SEAR countries to adopt a differential approach to further reduce maternal, newborn and child mortality based on their contextual priorities and available resources under the strategic leadership of the regional office.

Currently, there are a few gaps at the policy level, however with the emergence of new priorities, countries will need to adapt and adopt specific policies to address these issues. We observed that the population-based coverages of RMNCAH interventions have increased over time, but the increase has been uneven with large disparities within the countries. This is largely dependent upon the health systems-related constraints, especially health financing, human resources, and service delivery infrastructure. Efforts for improving access to healthcare services that include improved regional collaboration, capacity building programs, standardization of protocols and investing in skilled health workforce have long been underway for SEAR and need to be consolidated further.^37^ Promoting community engagement and empowerment is necessary and includes increasing awareness and education on maternal, newborn, and child health. The recent COVID-19 pandemic had an indirect impact on MNCH due to the disruption of provision and use of RMNCAH services. Significant disruptions were noted in services for family planning, antenatal care, institutional deliveries, postnatal care and treatment of acute childhood illnesses.^38^^,^^39^ Going forward, the countries should focus on building resilient primary health systems that are better prepared to withstand service disruption.^40^^,^^41^

The SEAR context offers challenges of resource constrainedness. Health financing has been a major challenge in this region; the total expenditure on health as percentage of GDP is as low as 2.63 in Bangladesh and 2.96 in India.^42^ It is lowest among all WHO regions in the world and highly variable across the member states.^43^ Additionally, financial protection is one of the key areas requiring urgent action and countries in the SEAR need to reduce out of pocket expenditures on health care.^44^ It is well known that an increase in healthcare financing helps in reduction of preventable maternal, newborn, child deaths and stillbirths.^45^

We found that higher maternal education, wealth index and urbanization were associated with improved coverage. Addressing country specific social determinants of health plays a significant role in improving maternal and child health. These include poverty, gender inequality, and social disparities.^46^^,^^47^ While these must be addressed, improving nutrition, sanitation, and hygiene practice are other areas which act as parallel arms to the health related indicators.^47^ In our analysis, women education and empowerment has been shown to impact health related outcomes for both mother and child, and hence member countries should strategize it for further leverage. However, this is unlikely without society-wide engagement and mobilization for which amplifying awareness through mass media and social media could be a possible options to explore.^48^ These efforts should not be limited to community-based stakeholders but also focus on building capacity and attitude of system-based providers as well.^49^ The Region has already released a monitoring framework across the life-course including gender, equity and rights approach.^50^ Such a framework will help in strengthening effective coverage of the programs.

With improved monitoring and evaluation data, program quality could be assessed and context-informed corrective measures undertaken.^51^ While most countries have defined monitoring frameworks and information systems in place at a national level for both maternal and child health, the barriers to collecting, collating and analyzing data at the ground level need to be addressed on a priority basis to achieve the related goals more effectively. There is an immediate need to focus on putting in functional surveillance systems that generate inter-operable data with regular updates at sub-national, national and regional levels for monitoring progress. The regional strategic framework for accelerating universal access to sexual and reproductive health in the WHO SEA Region 2020–2024 also highlights that triangulating data from the existing health management information systems has been challenging.^20^

Universal coverage of health-care services lays strong emphasis on good quality of care. It is, therefore, mandatory that interventions are delivered with sufficient quality, meeting appropriate standards of care. Our study found that all countries have established dedicated national cells, quality standards and forged strategic partnerships/alliances for quality of care. However the barriers to implementation of quality services at the ground level need to be addressed for further development and to achieve the positive health goals more effectively.^52^ POCQI is an effective programme tool developed to improve the health care practices with measurable results and can be used for health system strengthening.^53^ Effective coverage, i.e., coverage with quality, which is more representative of the actual ground level situation and is less liable to misinterpretation,^54^ needs to be measured instead of coverage reported only in terms of frequency or proportions achieved.

There are certain limitations in this article. National level data for regional averages was not available for few coverage indicators and we had to extract country data from different surveys and from different years, based on availability. Further, ARR has been used for projection of mortality rates for 2030 based on the assumption that all determinants and external factors remain constant through the given time periods, which might not be true in real time.

## Conclusion

The WHO SEAR has made progress in ending preventable mortality among mothers, newborns, and children, and is on track to achieve the SDG targets. The regional office provided leadership through instituting flagship for accelerating reduction in maternal, newborn and child mortality, regional committee resolution, and country-specific guidance and technical support to strengthen planning, implementation and monitoring under their national programmes. However, in addition to sustaining the gains, further actions are required by the region and the countries to address the challenges of widespread inequities, suboptimal quality of care in maternal, newborn and child health, emerging priorities like stillbirths, birth defects, early childhood development, and preparedness against emergencies like the current pandemic and climate change.

## Contributors

Neena Raina (NR): Conceptualization, Methodology, Writing-review and editing, Supervision, Resources.

Rajesh Khanna (RK): Conceptualization, Methodology, Writing—original draft, Writing-review and editing, Data curation, Formal analysis.

Shuchita Gupta (SG): Conceptualization, Methodology, Writing—original draft, Writing-review and editing, Data curation, Formal analysis.

Anoma Jayathilaka (AJ): Conceptualization, Methodology, Writing—original draft, Writing-review and editing, Data curation, Formal analysis.

Rajesh Mehta (RM): Conceptualization, Methodology, Writing—original draft.

Sabyasachi Behera (SB): Data curation, Formal analysis.

All authors contributed to writing, review and editing.

All authors contributed intellectual content and approved the final draft for publication.

All authors had full access to the data and take responsibility for the integrity and accuracy of the data analysis.

All authors had responsibility for the decision to submit the manuscript for publication.

## Declaration of interests

NR, RK, SG, and AJ are staff members, RM is WHO consultant and SB is freelance consultant. RM and SB declare no conflict of interest. The views expressed in the submitted article are of the authors and not an official position of the World Health Organization or any institution(s) to which they are affiliated.
