# Supplementary material for: Progress in achieving SDG targets for mortality reduction among mothers, newborns, and children in the WHO South-East Asia Region
Source: Lancet Reg Health Southeast Asia. 2023 Oct 29;18:100307. doi: 10.1016/j.lansea.2023.100307 (PMC10667297; doi:10.1016/j.lansea.2023.100307)
Supplement: Supplementary Tables S1–S4 [file mmc1.docx]

SUPPLEMENTARY MATERIAL

**Manuscript Title.** Progress in ending preventable mortality among mothers, newborns, and children toward achieving SDG targets in the WHO South-East Asia Region

Table of Contents

| **Supplementary Table 1: DHS/MICS surveys used for population-based coverage** | **:** | **2** |
| --- | --- | --- |
| **Supplementary Table 2: Presence of Key MNCH policy/guidelines in SEAR** | **:** | **2** |
| **Supplementary Table 3: Availability of TAG at the country-level** | **:** | **3** |
| **Supplementary Table 4: Effect of COVID-19 pandemic on MNCH indicators** | **:** | **4** |

Supplementary Table 1. DHS/MICS surveys used for population-based coverage

| **S.No.** | **Name of the country** | **Survey** | **Year** |
| --- | --- | --- | --- |
| 1 | Bangladesh | 1. Demographic and Health Survey | 1. 2014, 2017–2018 & 2022 |
| 2 | Bhutan | 1. Multiple Indicator Cluster Survey | 1. 2010 |
|  |  | 2. The State of the World's Children Reports | 2. 2019 |
|  |  | 3. Ministry of Health, National Health Survey | 3. 2012 |
| 3 | DPR Korea | 1. Multiple Indicator Cluster Survey | 1. 2009 & 2017 |
|  |  | 2. Demographic and Health Survey | 2. 2014 |
| 4 | India | 1. National Family Health Survey | 1. 2015–2016, & 2019–2021 |
| 5 | Indonesia | 1. Demographic and Health Survey | 1. 2012 & 2017 |
| 6 | Maldives | 1. Demographic and Health Survey | 1. 2009 & 2016–2017 |
| 7 | Myanmar | 1. Multiple Indicator Cluster Survey | 1. 2009–2010 |
|  |  | 2. Demographic and Health Survey | 2. 2015–2016 |
| 8 | Nepal | 1. Demographic and Health Survey | 1. 2016 & 2022 |
| 9 | Sri Lanka | 1. Demographic and Health Survey | 1. 2006–2007 & 2016 |
| 10 | Thailand | 1. Multiple Indicator Cluster Survey | 1. 2015–2016 & 2019 |
| 11 | Timor-Leste | 1. Demographic and Health Survey | 1. 2009–2010 & 2016 |

**Supplementary Table 2. Presence of key national policy/guidelines in maternal, newborn, and child health in WHO SEAR**

| **National-level policy/guideline** | BAN | BHU | DPRK | IND | INO | MAL | Myanmar | NEP | SRL | THA | TLS |
| --- | --- | --- | --- | --- | --- | --- | --- | --- | --- | --- | --- |
| Family planning/contraception | Yes | Yes | Yes | Yes | Yes | Yes | Yes | Yes | Yes | Yes | Yes |
| Antenatal care | Yes | Yes | Yes | Yes | Yes | Yes | Yes | Yes | Yes | Yes | Yes |
| Childbirth | Yes | Yes | Yes | Yes | Yes | No | Yes | Yes | Yes | Yes | Yes |
| Postnatal care for mothers and newborns | Yes | Yes | Yes | Yes | Yes | Yes | Yes | Yes | Yes | Yes | Yes |
| Management of low birth weight and preterm newborns | Yes | Yes | Yes | Yes | Yes | Yes | Yes | Yes | Yes | Yes | Yes |
| Child health and development of children | Yes | Yes | Yes | Yes | Yes | Yes | Yes | Yes | Yes | Yes | Yes |
| Early childhood development | Yes | Yes | Yes | Yes | Yes | Yes | No | Yes | Yes | Yes | No |
| Integrated management of childhood illness | Yes | Yes | Yes | Yes | Yes | Yes | Yes | Yes | Yes | Yes | Yes |
| Management of childhood pneumonia | Yes | Yes | Yes | Yes | Yes | Yes | Yes | Yes | Yes | Yes | Yes |
| Management of childhood diarrhoea | Yes | Yes | Yes | Yes | Yes | Yes | Yes | Yes | Yes | Yes | Yes |
| Management of malaria with appropriate recommendations for children | Yes | Yes | Yes | Yes | Yes | No | Yes | Yes | Yes | Yes | Yes |
| Management of acute malnutrition in children | Yes | Yes | Yes | Yes | Yes | Yes | Yes | Yes | Yes | Yes | Yes |

Supplementary Table 3. Availability of TAG at the country-level

| **Country** | **Status of national technical advisory group on RMNCAH** |
| --- | --- |
| BAN | No TAG on RMNCAH There are several working groups |
| BHU | No, Only TWG on CH |
| DPRK | No |
| IND | Yes, TWG also exists. |
| INO | No |
| MAL | No TAG, only RMNCAH coordination committee |
| Myanmar | Yes, RMNCAH Technical Strategic Group (TSG) exists. It is under the Myanmar Health Sector Coordination Committee (MHSCC), along with other TSGs. |
| NEP | Yes |
| SRL | Yes, National committee on FH and advisory committees |
| THA | No data |
| TLS | No TAG, only TWG |

Supplementary Table 4. Impact of COVID-19 pandemic on MNCH indicators for Bangladesh, Nepal, and Timor-Leste

| **Number of facility births** | **2019** | **2020** | **2021** | **% Change 2019-20** | **% Change 2019-21** | **% Change 2020-21** |
| --- | --- | --- | --- | --- | --- | --- |
| Bangladesh | 2617991 | 2383461 | 1915683 | -9.0 | -26.8 | -19.6 |
| Nepal | 423298 | 406715 | 430181 | -3.9 | 1.6 | 5.8 |
| Timor-Leste | 17199 | 16851 | 13612 | -2.0 | -20.9 | -19.2 |
|  |  |  |  |  |  |  |
|  |  |  |  |  |  |  |
| **Number of newborns breastfed within 1 hour of birth** | **2019** | **2020** | **2021** | **% Change 2019-20** | **% Change 2019-21** | **% Change 2020-21** |
| Bangladesh | 1305777 | 1001279 | 1094821 | -23.3 | -16.2 | 9.3 |
| Nepal | 502762 | 470906 | 415912 | -6.3 | -17.3 | -11.7 |
| Timor-Leste | NA | NA | NA | NA | NA | NA |
|  |  |  |  |  |  |  |
|  |  |  |  |  |  |  |
| **Number of children <5 years with diarrhoea treated in a facility** | **2019** | **2020** | **2021** | **% Change 2019-20** | **% Change 2019-21** | **% Change 2020-21** |
| Bangladesh | 929969 | 635182 | 713580 | -31.7 | -23.3 | 12.3 |
| Nepal | 358591 | 291974 | 321127 | -18.6 | -10.4 | 10.0 |
| Timor-Leste | 36955 | 30063 | NA | -18.6 | NA | NA |
|  |  |  |  |  |  |  |
|  |  |  |  |  |  |  |
| **Number of newborns who received care within 24 hours of birth** | **2019** | **2020** | **2021** | **% Change 2019-20** | **% Change 2019-21** | **% Change 2020-21** |
| Bangladesh | 1360308 | 1088411 | 1087275 | -20.0 | -20.1 | -0.1 |
| Nepal | 380533 | 379046 | 374643 | -0.4 | -1.5 | -1.2 |
| Timor-Leste | 8342 | 5061 | 14759 | -39.3 | 76.9 | 191.6 |
